# Lack of STAT6 enhances murine acute lung injury through NLRP3/p38 MAPK signaling pathway in macrophages

**DOI:** 10.1186/s12865-022-00500-9

**Published:** 2022-05-23

**Authors:** Lu Hu, Changzhou Shao, Linyue Pan, Zhilong Jiang

**Affiliations:** 1 Department of Pulmonary Medicine, Zhongshan Hospital Fudan University, 180 Feng Lin Road, Shanghai, 200032 China; 2 Department of Respiratory, The Second Affiliated Hospital of Xi’an Jiaotong University, Xi’An, China

**Keywords:** Acute lung injury, Macrophages, Pyroptosis, Cytokines, STAT6

## Abstract

**Background:**

Signal transducer and activator of transcription 6 (STAT6) is an intracelluar transcriotion factor and NLRP3 (Nod-like receptor containing a pyrin domain 3) is a component of NLRP3 inflammasome in pyroptotic cells. There was increased activation of STAT6 and expression of NLRP3 in mice with murine acute lung injury (ALI). However, it is unknown their roles in the development of murine ALI. We in this study, investigated the effects of STAT6 signaling on murine ALI and pyroptosis in STAT6 knock-out (KO) mice and macrophages.

**Results:**

STAT6 was activated in the lung tissues of mice 2 days after intratracheal treatmemt with 5 mg/kg LPS. Lack of STAT6 expression in KO mice induced more severe lung inflammation, associated with elevated neutrophil influx and expression of TNF-alpha, IL-6 and IL-1beta in the inflamed lung tissues. In addition, the expression of NLRP3, ASC (apoptosis-associated speck-like protein containing a CARD), p-p38 MAPK (p38 mitogen-activated protein kinase) and ratio of LC3-II/I (microtubule-associated protein-1 light chain-3) was increased, accompanied with the increased polarization of Siglec-F(−) subtype macrophages in KO mice with ALI. Further studies in bone marrow-derived macrophages (BMDMs) revealed that lack of STAT6 increased the expression of NLRP3 and p-p38 MAPK, in association with elevated expression of TNF-alpha, IL-1beta and Calreticulin in LPS-treated KO BMDMs.

**Conclusions:**

Lack of STAT6 exacerbated murine ALI through improving the expression of NLRP3 and activation of p38 MAPK in macrophages. STAT6 has an immune suppressive role in the development of ALI and would be a promising therapeutic target in the treatment of ALI and possibly among patients with acute respiratory distress syndrome (ARDS).

**Supplementary Information:**

The online version contains supplementary material available at 10.1186/s12865-022-00500-9.

## Inrtoduction

STAT6 is an intracellular transcription factor and plays an important role in cell proliferation and differentiation [1, 2]. Recent studies showed that STAT6 is critically involved in asthma and lung fibrosis. Suppression of STAT6 activity resulted in lower airway hyper-responsiveness and lung eosinophilia in mice after respiratory syncytial virus reinfection [3]. In STAT6 knock-out (KO) asthmatic mice, the expression of IL-4 and IL-13 was up-regulated in eosinophils and that induced more population of Th2 cells [4, 5]. Thus, STAT6 participates in Th2 immune responses in asthma. In addition, inhibition of STAT6 signaling by STAT6 inhibitor AS1517499 significantly enhanced the development of lung fibrosis, associated with reduced macrophage efferocytosis and elevated expression of pro-inflammatory cytokines. In contrast, activation of STAT6 signaling attenuated lung fibrosis, with improved macrophage phagocytosis and lung inflammation resolution [6]. Thus, STAT6 has a protective role in lung fibrosis through facilitating macrophage phagocytosis [7, 8].

Recent studies also showed the critical role of STAT6 in the development of acute lung injury (ALI) in mice and acute respiratory distress syndrome (ARDS) in patents [9, 10]. For example, lack of STAT6 expression increased pancreatitis-associated lung injury and the expression of myeloperoxidase [11]. Additional studies also revealed the role of STAT6 signaling in IL-4 and MCTR1 (an endogenously pro-resolution lipid mediator)-mediated therapy of ALI [9, 12]. Our previous study also showed that Calreticulin neutralizing antibody (aCALR) can suppress ALI through STAT6 signaling in macrophages. Lack of STAT6 expression abolished the therapeutic effects of aCALR, indicating the immune regulatory role of STAT6 in macrophage function [10].

It was previously reported that TNF-alpha, IL-1beta, IL-18 and IL-6 were highly expressed in mice with ALI [10, 13]. IL-1beta and IL-18 are exclusively produced by pyroptotic macrophages, indicating the involvement of pyroptosis in LPS-induced ALI. Under oxidative stress, NLRP3 inflammasome containing NLRP3 (Nod-like receptor containing a pyrin domain 3), ASC (apoptosis-associated speck-like protein containing a CARD) and pro-Caspase-1 is formed and activated in pyroptotic macrophages, subsequently releasing a large amount of active pro-inflammatory cytokines, IL-1beta and IL-18 [14, 15]. LPS and other insulants are inducer of NLRP3 inflammasome formation [16]. Though the expression of NLRP3 and activation of STAT6 were elevated in mouse model with ALI [17], it remains unclear whether LPS drives formation of NLRP3 inflammasome and macrophage pyroptosis through STAT6 signaling. To address this issue, we in this study, treated wild-type (WT) and STAT6 KO mice with LPS. The results showed that lack of STAT6 in KO mice induced more severe ALI, associated with higher expression of NLRP3, activation of p38 MAPK (p38 mitogen-activated protein kinase) and production of pro-inflammatory cytokines than those in WT mice. Therefore, STAT6 signaling is protective in ALI through suppressing NLRP3/p38 MAPK signaling pathway.

## Methods

### Mice and treatment

STAT6 KO mice on the C57BL/6 background were obtained from the Nanjing Model Animal Center, in which the SH2 domain of STAT6 gene was replaced with a neomycin resistance (neor) cassette. The phenotype of STAT6 KO mice were identified by PCR method. The primer sequences were as follows: (1) Stat6IMR0092: 5′-AATCCATCTTGTTCAATGGCCGATC-3′; (2) Stat6IMR1822: 5′-ACTCCGGAAAGCCTCATCTT-3′; (3) Stat6IMR7416: 5′-AAGTGGGTCCCCTTCACTCT-3′. 380 bp and 280 bp PCR products were identified as wild-type and mutant STAT6 genes respectively. 8–10 weeks old of WT and KO male mice were intratracheal (i.t.) injected with 5 mg/kg lipopolysaccharides (LPS) from Escherichia coli O55:B5 (Sigma-Aldrich, St Louis, MO) for 2 days, according to our previously published protocol [18]. The mice treated with PBS were used as controls. Bronchoalveolar lavage (BAL) and lung tissues were collected for analysis. All animals were housed and treated under the guidelines of the Institutional Animal Care and Use Committee of the Fudan University, Zhongshan Hospital in China. All experiments were approved by the committee and performed in the Zhongshan Hospital, Fudan University.

### Culture and treatment of BMDMs

Bone marrow cells were flushed from the femurs and tibiae of mice and cultured in RPMI1640 culture medium supplied with 10% fetal bovine serum (FBS) and 20% conditional media of NIH3T3 cells for 6 days to obtain bone marrow-derived macrophages (BMDMs). BMDMs from WT and KO mice were stimulated with 500 ng/ml LPS for 24 h, according to our previously published protocol [10]. The untreated cells were used as controls. The treated cells and supernatants were analyzed for protein expression by flow cytometry, immunostaining, Western blot and ELISA assay.

### Western blot analysis

The protein expression of total STAT6, NLRP3, ASC, total p38 MAPK, p-p38 MAPK (Thr180/Tyr182), p62 and LC3 in the lung tissues and cells were analyzed by Western blot analysis. Primary antibodies included rabbit anti-total STAT6 (Bioss antibodies, Boston, MA), rabbit anti-ASC and rabbit anti-NLRP3 (Abcam, Cambridge, MA), rabbit anti-p-p38 MAPK, rabbit anti-p62 and rabbit anti-LC3 (Cell signaling technology, Danvers, MA). The anti-mouse glyceraldehyde-3-phosphate dehydrogenase (GAPDH) or β-Tubulin antibodies were used as loading internal controls. Protein expression was quantitatively analyzed by ImageJ software and data was presented as ratio of densitometric density of target protein to internal loading controls.

### Immunostaining assay

The expression of p-STAT6 (Tyr641), NLRP3, ASC, p-p38 MAPK in the lung tissues of mice and/or macrophages were analyzed by immunostaining assay. Briefly, the lung tissue sections and cells were fixed with 4% paraformaldehyde (4% PFA). After incubation with 0.05% Triton X-100 and 10% goat serum, the sections or cells were incubated with primary antibody (dilution 1:200) for 3 h and followed by incubation with Cy3 or FITC-conjugated anti-rabbit IgG (dilution 1:500) for 1 h. Nuclei were stained by 4′,6-diamidino-2-phenylindole (DAPI). After washed with PBS for 3 times, the stained sections or cells were visualized under fluorescence microscope. The positively stained cells and sections were quantitatively analyzed by ImageJ software or manual counting positively stained cells and total nuclei in one field. Data was presented as percentage of positively stained cells to total nuclei or ratio of fluorescent intensity to control group.

### ELISA assay

The concentration of TNF-alpha, IL-6, IL-1beta and Calreticulin in BAL or cell supernatants were measured by ELISA assay according to industrial instructions (R&D systems, Minneapolis, MN).

### Flow cytometry analysis

1 × 10^6^ cell suspension from lung digests and BAL were incubated with antibody cocktail containing APC-anti-CD206 (BioLegend. San Diego, CA), PerC-Cy5-anti-F4/80, PE-Cy7-anti-Ly6G, PE-Cy3-anti-p-STAT6, APC-Cy7-anti-CD11b, BV421-anti-Siglec-F (BD Biosciences, Franklin Lakes, NJ and eBioscience, San Diego, CA). After incubation at dark for 40 min, the stained cells were washed for 2 times and analyzed on FACScan cytometer and Flow Jo software, version 8.8.4 (Becton, Dickinson and Company, Franklin Lakes, NJ).

### Statistical analysis

Results are presented as mean ± standard error of each group. All data were analyzed by Student’s t test for comparison between two groups and one-way analysis of variance (ANOVA) followed by Tukey’s multiple comparisons test for over two groups. A value of *p* < 0.05 was considered as statistically significantly different.

## Results

### STAT6 was activated in mice with LPS-induced ALI

**Fig. 1:**
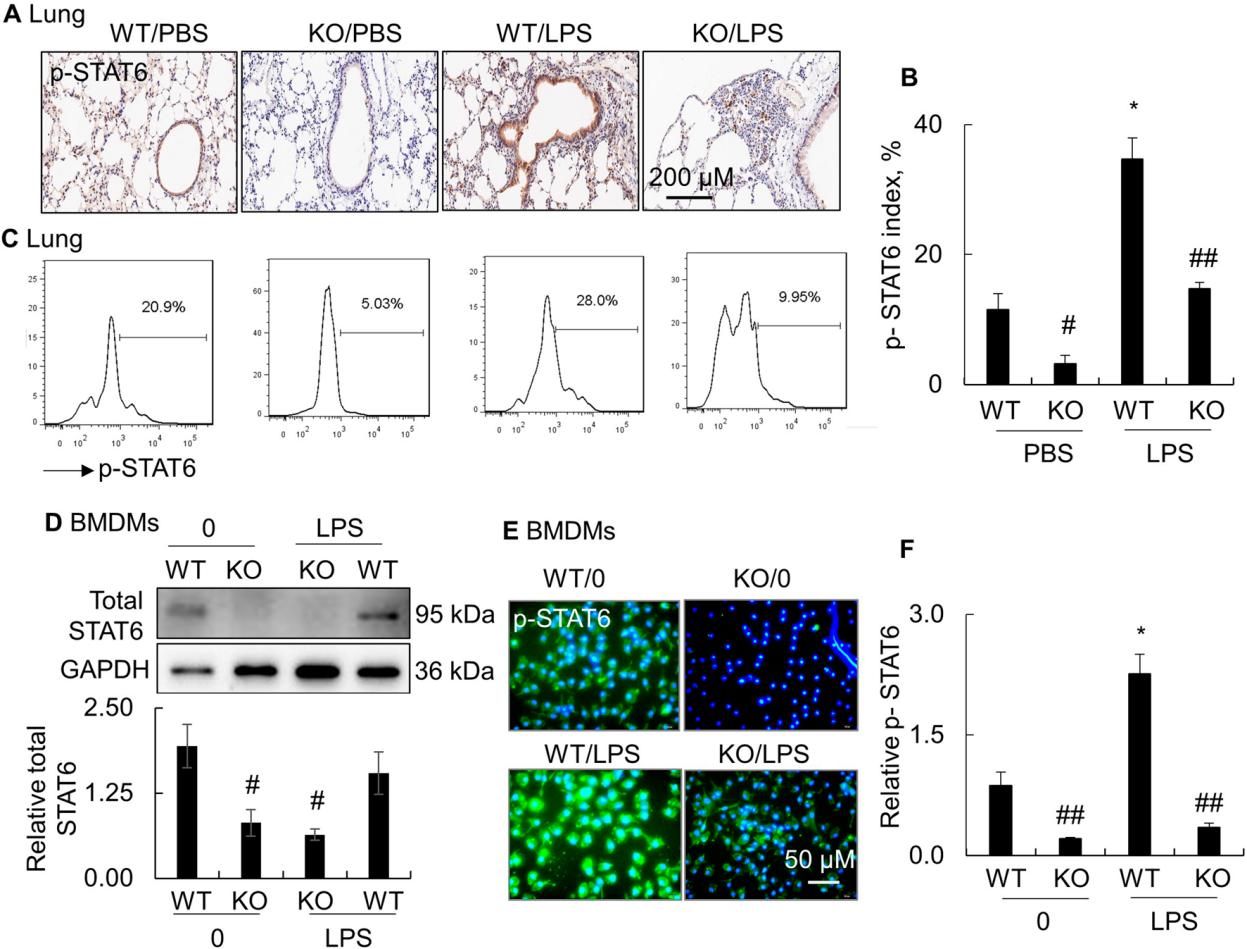
STAT6 was activated in LPS-treated mice and macrophages. Wild type (WT) and STAT6 knock-out (KO) adult mice were intratracheal (i.t.) injected with 5 mg/kg LPS or PBS for 2 days. **A** Immunostaining for phosphorylated STAT6 (p-STAT6) in the lung tissues of treated mice. Representative photograph with 100 × magnification. **B** Quantitative analysis of p-STAT6 + cells in lung tissues after immunostaining. The total number of nuclei and those in p-STAT6 + cells were scored. At least 8 fields per sample were counted at 200 × magnification. Data was presentd as the percentage of nuclei in p-STAT6 + cells to total nuclei in one field. **C** Representative histogram of p-STAT6 + cells in the lung digests by flow cytometry. **D** Western blot analysis for the expression of total STAT6 in LPS-treated bone marrow-derived macrophages (BMDMs). GAPDH is internal loading control (upper panel). Quantitative analysis of toal STAT6 expression in BMDMs (lower panel). Data was presented as ratio of STAT6 densitometric density to GAPDH. **E** Immunostaining for p-STAT6 in BMDMs. Green: positively stained cells; Blue: DAPI-stained nuclei. Representative photograph with 200 × magnification. **F** Quantitative analysis of fluorescence intensity of p-STAT6 + cells by ImageJ software after immunostaining. Data was presented as ratio of fluorescence intensity to WT/0 group. All quantitative data was presented as mean ± standard error, n = 3–7, *p < 0.05 versus the untreated controls; #*p* < 0.05, ##*p* < 0.01 versus WT group. One-way ANOVA test followed by Tukey’s multiple comparisons

In murine ALI, we found that the phosphorylated STAT6 (p-STAT6) was increased in the lung tissues of LPS-treated wild-type (WT) mice, compared to the PBS-treated control mice (brown). However, p-STAT6 was not well detected in the lung tissues of STAT6 knock-out (KO) mice, indicating lack of STAT6 activity in KO mice (Fig. 1A, B). The effects of LPS on STAT6 activation were further confirmed by flow cytometry analysis, in which LPS moderately increased the percentage of p-STAT6 + cells in the lung tissues of WT mice. However, p-STAT6 + cells were low or undetectable in KO mice (Fig. 1C). Additional study in bone marrow-derived macrophages (BMDMs) also showed that total STAT6 was well detected in WT BMDMs, but not in KO BMDMs, indicating lack of STAT6 expression in KO BMDMs (Fig. 1D). Furthermore, we observed the largely increased fluorescence intensity of p-STAT6 + cells (green) in LPS-treated WT BMDMs, compared to the untreated cells. However, p-STAT6 was not well detected in KO BMDMs by immunostaining (Fig. 1E, F). The results showed the effects of LPS in activation of STAT6, that encouraged us to further investigate the role of STAT6 in the development of ALI.

### Lack of STAT6 expression in STAT6 KO mice induced more severe ALI and increased the expression of pro-inflammatory cytokines

**Fig. 2:**
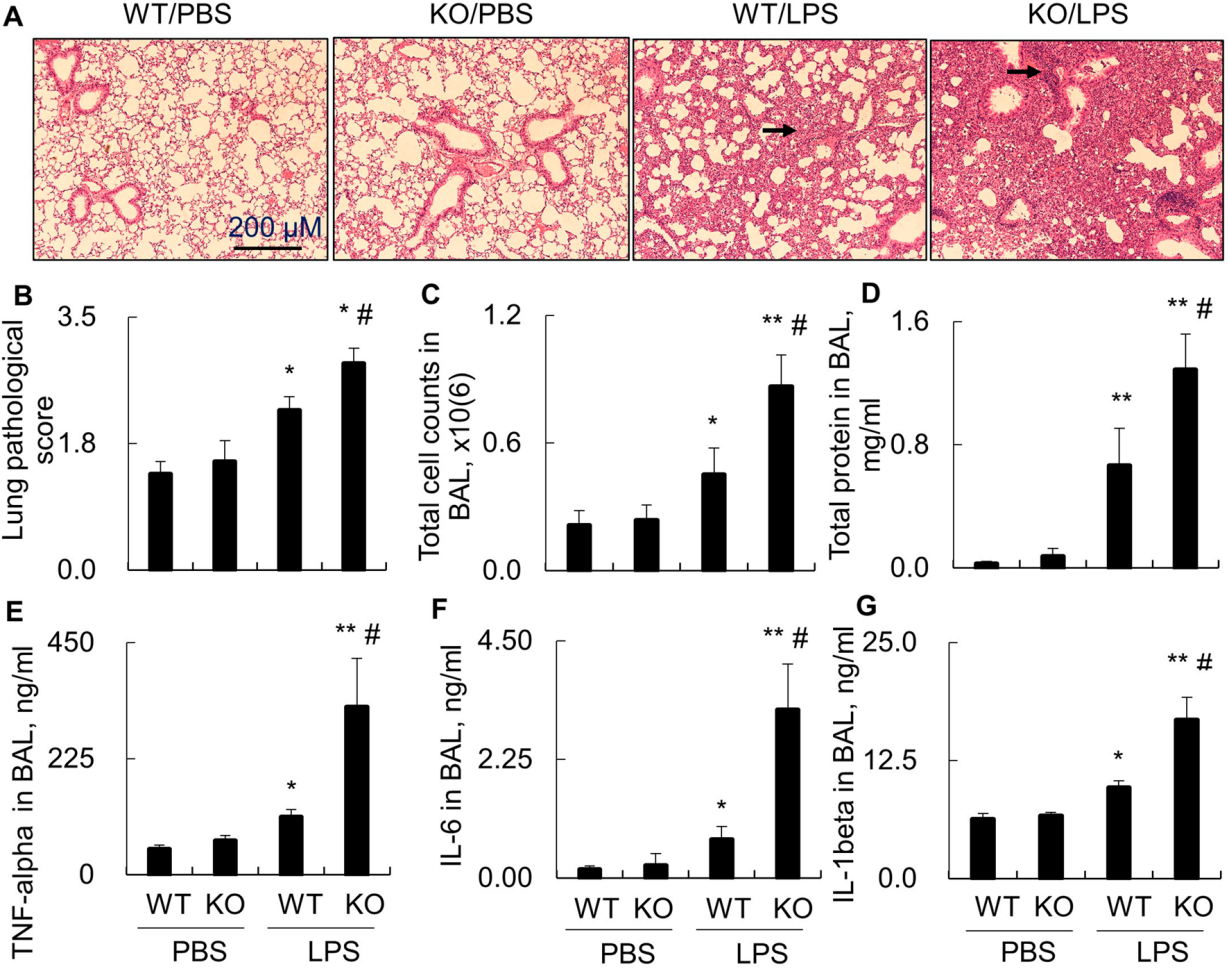
Lack of STAT6 expression in KO mice induced more severe ALI. **A** Lung histology of WT and KO mice with LPS-induced ALI by H&E staining. Representative photograph with 100× magnification. Black arrows indicated the inflammatory infiltrates. **B** Quantitative analysis of lung inflammation in each group of mice. Lung injury severity was evaluated by scale from 0 to 4 in terms of infiltrating inflammatory cells and alveoli destruction. **C** Total cell counts in BAL. **D** Total protein content in BAL by BCA assay. **E**–**G** ELISA analysis for the expression of TNF-alpha, IL-6 and IL-1beta in BAL. All quantitative data was presented as mean ± standard error, *p < 0.05, **p < 0.01 versus PBS group, #*p* < 0.05 versus WT/LPS group, n = 3–7. One-way ANOVA test followed by Tukey’s multiple comparisons

STAT6 was activated in LPS-induced murine ALI. To further investigate whether STAT6 participates in the development of murine ALI, we i.t. treated WT and KO mice with 5 mg/kg LPS for 2 days. Analysis of the collected lung tissues indicated that LPS largely increased acute lung inflammation in WT mice. Lack of STAT6 expression in LPS-treated KO mice induced more severe acute lung inflammation than that in LPS-treated WT mice (Fig. 2A, black arrow). The lung pathological score (Fig. 2B), total cell counts (Fig. 2C) and protein content (Fig. 2D) in BAL were significantly increased in LPS-treated KO mice, compared to the LPS-treated WT mice. Consistently, lack of STAT6 significantly increased the expression of pro-inflammatory cytokines, including TNF-alpha, IL-6 and IL-1beta in BAL of LPS-treated KO mice, compared to the LPS-treated WT mice, indicating the protective role of STAT6 in murine ALI (Fig. 2E–G).

### Lack of STAT6 expression in STAT6 KO mice increased the expression of NLRP3, ASC and p-p38 MAPK

**Fig. 3:**
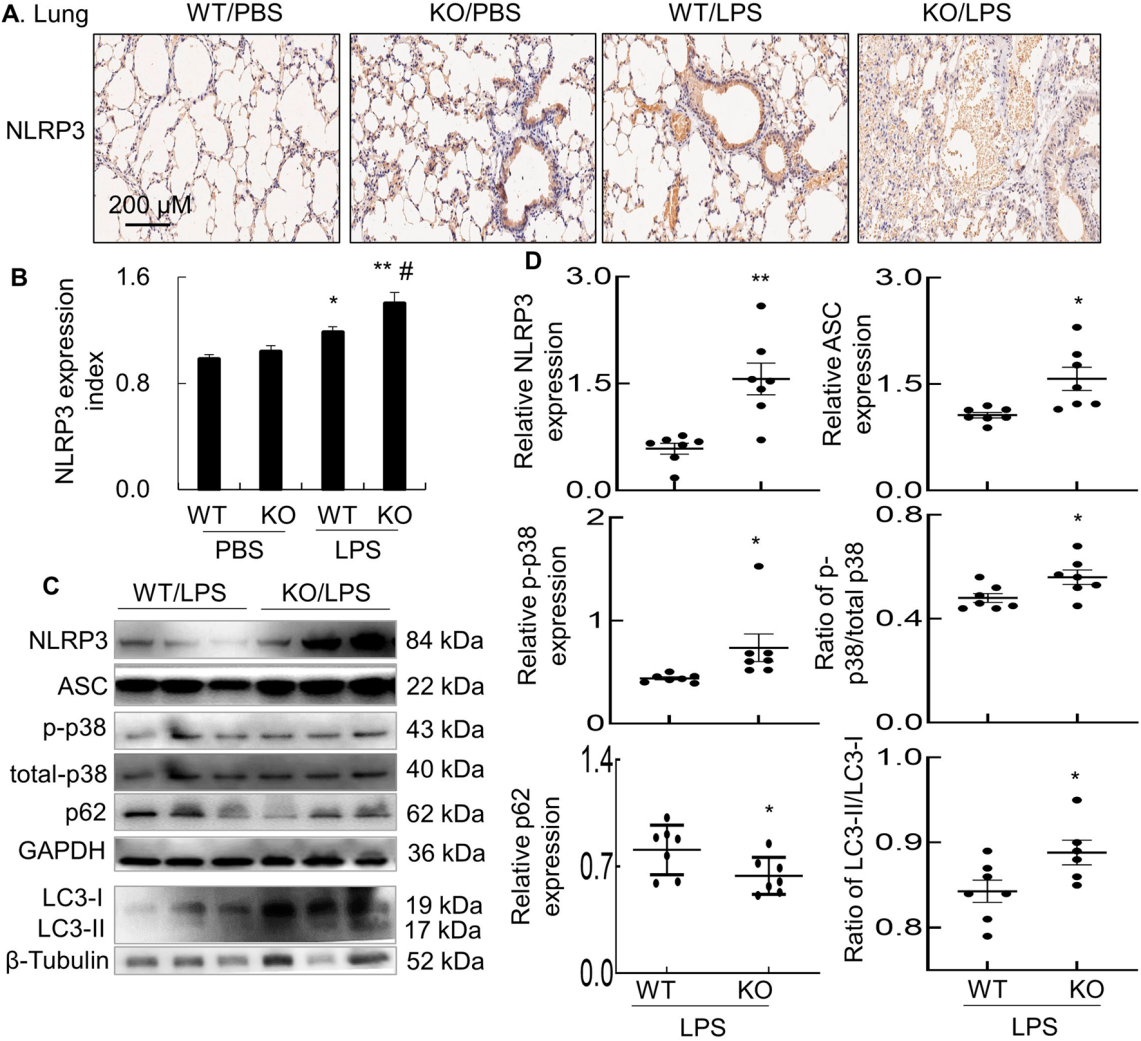
Lack of STAT6 expression in KO mice induced more expression of NLRP3 and activation of p38 MAPK. **A** Immunostaining for NLRP3 expression in the lung tissues of treated mice with ALI. Representative photograph with 100 × magnification. Brown: positively stained cells. **B** Quantitative analysis for NLRP3 expression in the lung tissues of mice after immunostaining by ImageJ software. Data was presented as mean ratio of NLRP3 staining density to WT/PBS control ± standard error. n = 3–7, *p < 0.05, **p < 0.01 versus PBS group. # *p* < 0.05 versus WT/LPS group. One-way ANOVA test followed by Tukey’s multiple comparisons. **C** Western blot analysis for the expression of NLRP3, ASC, p-p38 MAPK, total p38 MAPK, p62 and LC3 in the lung tissues of mice with ALI. GAPDH and β-Tubulin are internal loading controls. Representative blots. **D** Quantitative analysis for the expression of NLRP3, ASC, p-p38 MAPK, ratio of p-p38 MAPK/total p38 MAPK, p62 and LC3 in Western blots by ImageJ software. Data was presented as ratio of densitometric density to GAPDH or β-Tubulin. *p < 0.05, **p < 0.01 versus WT group. Student *t* test

NLRP3 is a key intracellular protein involved in cell pyroptosis. After cell activation, NLRP3 is up-regulated and forms NLRP3 inflammasome complex, subsequently releasing active inflammatory cytokines IL-1beta and IL-18 [14, 15, 19]. However, it remains unknown whether cell pyroptosis is involved in the development of ALI. To address this issue, we analyzed the expression of NLRP3 in the inflamed lung tissues of murine ALI by immunostaining. The results revealed that there was increased expression of NLRP3 (brown) in murine ALI and lack of STAT6 expression futher increased the expression of NLRP3, compared to the mice of WT/LPS group (Fig. 3A, B). The results were further confirmed by Western blot analysis, in which a higher expression of NLRP3 and ASC was observed in the lung tissues of KO mice with ALI, than those in WT mice (Fig. 3C, D). It was previously reported that p38 MAPK signaling pathway was positively involved in cell pyroptosis [17] and p62 expression was negatively associated with cell autophagy [20, 21]. Our further analysis by Western blotting displayed a more phosphorylated p38 MAPK and lower expression of p62 in the lung tissues of KO mice than those in WT mice (Fig. 3C, upper panel and D; Additional file 2: Fig. S2).

LC3 is involved in formation of autophagosomes during cell autophagy [20]. Our additional study by Western blotting also revealed more expression of LC3-II and higher ratio od LC3-II/LC3-I in the lung tissues of KO mice than those in WT mice (Fig. 3C, lower panel and D). These results indicated more cell autophagy in the lung tissues of KO mice than WT mice after LPS treatment. Thus, lack of STAT6 expression increased both cell pyroptosis and autophagy in the inflamed lung tissues of mice with ALI. p38 MAPK signaling pathway was involved in the increased cell pyroptosis and autophagy.

### Siglec-F(−) subtype macrophages were increased in STAT6 KO mice with ALI

**Fig. 4:**
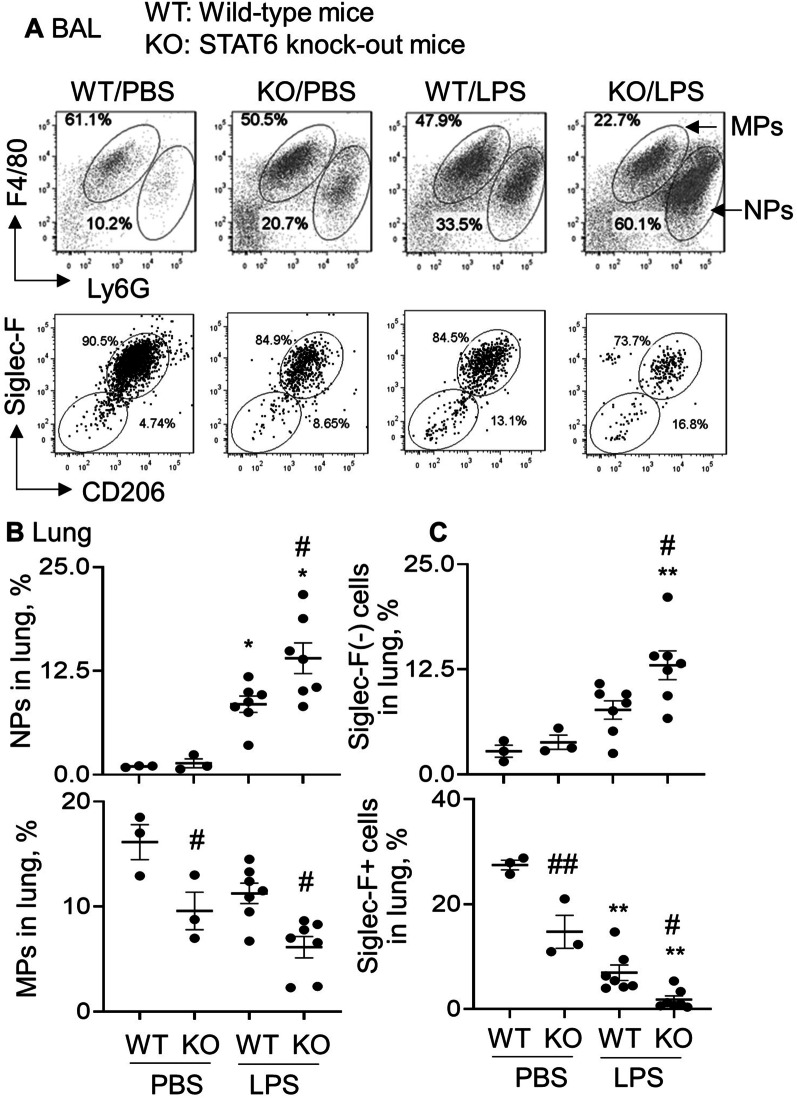
Lack of STAT6 expression in KO mice induced polarization of Siglec-F(−) subtype macrophages. **A** Flow cytometry analysis for the percentage of F4/80(low)Ly6G(high) neutrophils (NPs) and F4/80(high)Ly6G(low) macrophages (MPs) in BAL of murine ALI (upper panel). Siglec-F(−) and Siglec-F + subtype macrophages were gated on MPs (lower panel). Representative dot plot in each group. **B** Quantitative analysis for the percentage of NPs and MPs in the lung tissues of murine ALI after flow cytometry analysis. **C** Quantitative analysis for the percentage of Siglec-F(−) and Siglec-F + subtype macrophages in the lung tissues of murine ALI. All quantitative data was presented individual data ponts, *p < 0.05, **p < 0.01 versus PBS group; #*p* < 0.05, ##*p* < 0.01 versus WT group. One-way ANOVA test followed by Tukey’s multiple comparisons

It was previously reported that macrophages were divided into Siglec-F(−) and Siglec-F + subtypes [10]. To investigate whether lack of STAT6 expression in KO mice affected polarization of macrophages into the subtypes, we analayzed the expression of Siglec-F in F4/80(high)Ly6G(low) macrophages (MPs) and F4/80(low)Ly6G(high) neutrophils (NPs) by flow cytometry analysis. The results revealed that the population of NPs were significantly increased in the lung tissues and BAL of WT mice with ALI. Lack of STAT6 expression in KO mice induced twofold more NPs influx into BAL and lung tissues than those in WT mice with ALI (Fig. 4A, B upper panels). Due to massive influx of NPs into the lung tissues and BAL, the percentage of MPs were relatively lower in the KO mice, compared to the WT mice (Fig. 4A, upper panel and 4B, lower panel). Further analysis of MP subtypes revealed that the percentage of Siglec-F(−) subtype macrophages was increased and the percentage of Siglec-F + subtype macrophages was decreased in BAL and lung tissues of LPS-treated WT mice, compared to the PBS-treated WT control mice. Lack of STAT6 expression in KO mice induced more percentage of Siglec-F(−) subtype macrophages and lower percentage of Siglec-F + subtype macrophages than those in WT mice (Fig. 4A, lower panel and 4C). Subsequently, the ratio of Siglec-F(−)/Siglec-F + subtype macrophages was increased in the lung tissues of KO mice (data not shown). Therefore, lack of STAT6 increased severity of ALI, associated with Siglec-F(−) subtype macrophage-biased polarization in vivo.

### Lack of STAT6 increased NLRP3/p38 MAPK signaling and the expression of pro-inflammatory cytokines in bone marrow-derived macrophages (BMDMs)

**Fig. 5:**
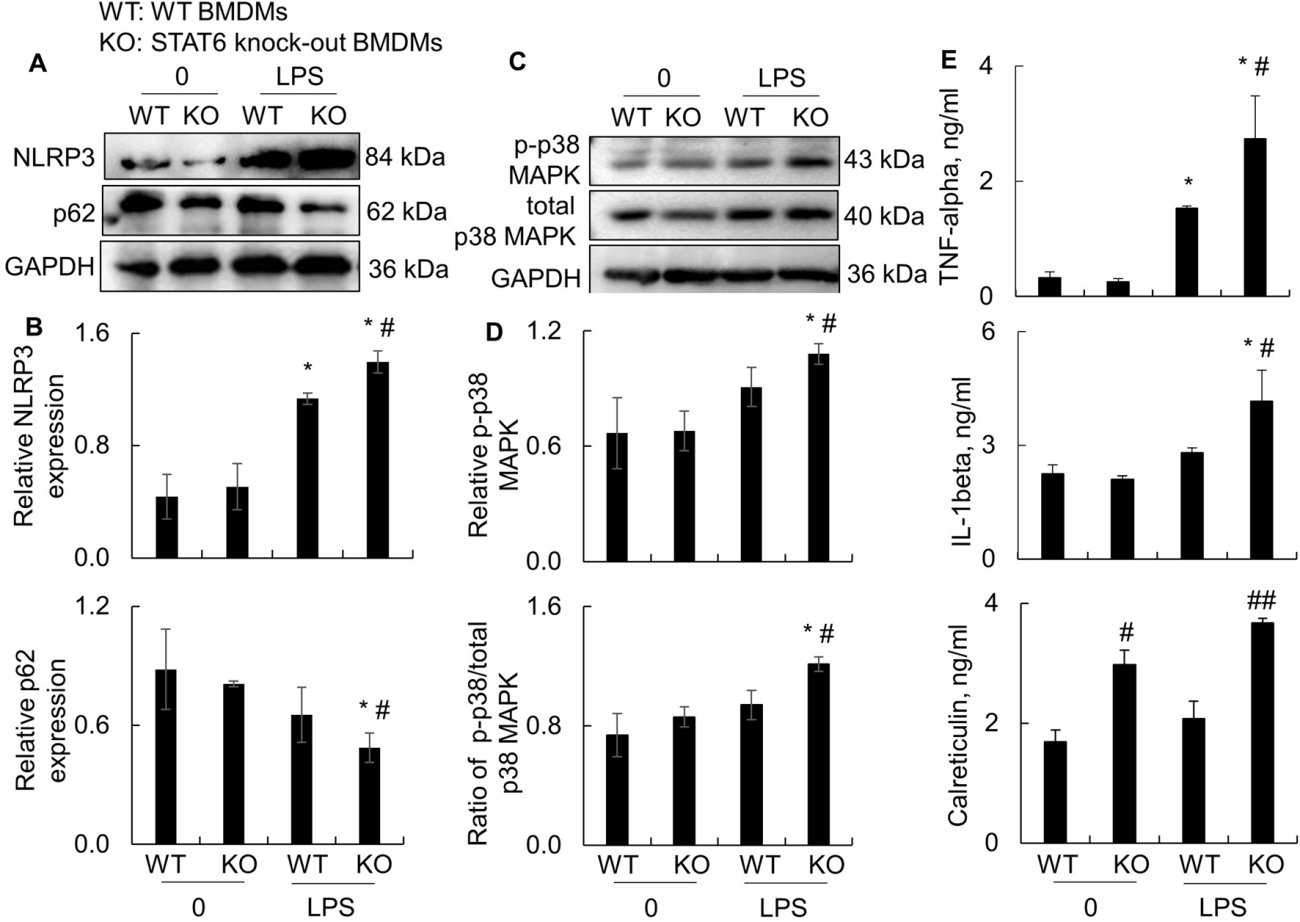
Lack of STAT6 increased the expression of NLRP3 and pro-inflammatory cytokines in bone marrow-derived macrophages (BMDMs). BMDMs from WT and STAT6 KO mice were treated with or without 500 ng/ml LPS for 24 h. **A** Western blot analysis for the expression of NLRP3 and p62 in the treated WT and KO BMDMs. **B** Quantitative analysis for the expression of NLRP3 and p62 in Western blots by ImageJ software. **C** Western blot analysis for p-p38 MAPK and total p38 MAPK in the treated WT and KO BMDMs. **D** Quantitative analysis for p-p38 MAPK and ratio of p-p38 MAPK/total p38 MAPK in Western blots by ImageJ software. Data was presented as ratio of densitometric density to GAPDH internal loading control. **E** ELISA analysis for the expression of TNF-alpha, IL-1beta and Calreticulin in the supernatants of treated cells. All quantitative data was presented as mean ± standard error. *p < 0.05 versus untreated group; #*p* < 0.05, ##*p* < 0.01 versus WT group, n = 3–4. One-way ANOVA test followed by Tukey’s multiple comparisons

To further define whether the increased severity of ALI in KO mice was associated with the improved pyroptosis in macrophages, we treated BMDMs from WT and KO mice with LPS for 24 h. Western blot analysis showed that LPS treatment significantly increased the expression of NLRP3 and reduced the expression of p62 in WT BMDMs, that was further altered in LPS-treated KO BMDMs (Fig. 5A, B; Additioanl file 3: Fig. S3). In addition, we observed the elevated p-p38 MAPK and increased ratio of p-p38 MAPK/total p38 MAPK in KO BMDMs, compared to the WT BMDMs (Fig. 5C, D). Furthermore, the effects of STAT6 expression on NLRP3 and p-p38 MAPK were analyzed by immunostaining, in which we observed more expression of NLRP3, ASC and p-p38 MAPK in LPS-treated KO BMDMs than those in LPS-treated WT BMDMs (Additional file 1: Fig. S1), indicating that STAT6 has suppressive role in macrophage pyroptosis and activation of p38 MAPK. Consistent with the improved macrophage pyroptosis in LPS-treated KO BMDMs, we observed more expression of pro-inflammatory cytokines and mediators, including TNF-alpha, IL-1beta and Calreticulin in the LPS-treated KO BMDMs than those in LPS-treated WT BMDMs (Fig. 5E). Therfore, lack of STAT6 induced more severe ALI possibly through increasing p-p38 MAPK signaling-mediated macrophage pyroptosis and autophagy.

## Discussion

Pyroptotic macrophages are featured by formation of NLRP3 inflammasome and release of active pro-inflammatoy cytokines, IL-18 and IL-1beta. In addition, Calreticulin is highly expressed in the apoptotic macrophages, facilitating clear-up of the apoptotic cells by phagocytes [22]. It was previously reported that NLRP3, IL-18, IL-1beta and Calreticulin significantly contributed to the development of ALI in mice [17]. Thus, suppression of pyroptotic macrophages through molecular intervention might be an effective therapeutic strategy in the treatment of ALI and patients with ARDS [23–25].

It is documented that pro-inflammatory microenvironment may contribute to the high expression of NLRP3, because previous report showed that NLRP3 expression was up-regulated by pro-inflammatory cytokines, TNF-alpha and IL-6 [10]. In addition, the expression of NLRP3 was increased by potassium ionophore nigericin and ATP [26], and suppressed by NLRP3 inhibitor (Glibenclamide), Rho-associated coiled-coil kinase inhibitor (Y-27632) and Caspase-1 inhibitor [16]. However, it remains unclear whether STAT6 signaling pathway is involved in the expression and formation of NLRP3 inflammasome and development of ALI in mice.

In a mouse model with ALI, we observed more expression of NLRP3 and activation of STAT6 signaling. To define whether macrophage pyroptosis contributes to the development of ALI through STAT6 signaling, we established a mouse model with ALI in WT and STAT6 KO mice. The results showed that lack of STAT6 in KO mice induced more severe ALI, accompanied with elevated infiltrating neutrophils and expression of TNF-alpha, IL-6 and IL-1beta. The results indicated the immune regulatory role of STAT6 signaling in the mice with ALI. Further analysis of macrophage subtypes revealed the increased ratio of Siglec-F(−)/Siglec-F + subtype macrophages in KO mice with ALI. The results confirmed the role of STAT6 signaling in promoting possible anti-inflammatory Siglec-F + subtype macrophages, supporting the previous reports [27, 28]. Thus, lack of STAT6 in KO mice increased severity of ALI, associated with increased Siglec-F(−) subtype macrophages.

In addition to the increased Siglec-F(−) subtype macrophages in KO mice, we also observed more pyroptotic macrophages in KO mice with ALI, as evidenced by increased expression of NLRP3, ASC and p-p38 MAPK in the lung tissues of KO mice. Further study in BMDMs also showed the increased expression of IL-1beta, TNF-alpha and Calreticulin in KO BMDMs. Thereby, lack of STAT6 increased ALI severity, possibly through increasing both Siglec-F(−) subtype macrophages and subsequently improving release of pro-inflammatory cytokines and mediators. According to the previously report, STAT6 signaling facilitated macrophage phagocytosis [6], we speculate that the reduced macrophage phagocytosis activity and clearance of dead cells in KO mice maybe involved in increased severity of ALI in mice, that would be investigated in the future. In addition, it is worth mentioning that the study was limited by using conventional STAT6 KO mice, in which STAT6 gene expression is defective in all type of cells. The expression and activation of STAT6 in other cell types, such as lung epithelial cells may contribute to the development of ALI. Therefore, myeloid cell (Lyz2/Cre-STAT6/loxp) and lung epithelial cell (SP-C/Cre-STAT6/loxp)-conditional knockout mice, should be used in our future study to define the role of macrophage and lung epithelial cell-specific STAT6 signaling in murine ALI.

Though we found the protective role of STAT6 signaling in murine ALI, it remains unclear what STAT6 downstream signaling pathways were involved in the suppression of ALI. Previous report showed that LPS induced NLRP3 inflammasome activation through p38 MAPK signaling pathway [22]. Consistent with the previous report, we observed the increased p-p38 MAPK in wild-type of murine ALI and LPS-treated macrophages, and the effects were further enhanced in STAT6-deficient murine ALI and macrophages, confirming the suppressive role of STAT6 signaling in p38 MAPK activation in vivo and in vitro. The results supported the previous report, in which IL-13 suppressed p38 MAPK activation through activation of STAT6 signaling in intestine epithelial cells [29]. However, a controversial report indicated the promoting role of STAT6 in p38 MAPK signaling, as evidenced by failure to inducing activation of p38 MAPK in tracheal epithelial cells of STAT6 KO mice after IL-13 treatment [30]. The descripancy maybe caused by different type of cells and animal models, it remains to be clarified in the future. Taken together, we in this study conclude that lack of STAT6 enhanced murine ALI severity, that was associated with increased macrophage pyroptosis and subsequent expression of pro-inflammatory cytokines and mediators. Thereby, STAT6 signaling has anti-inflammatory and protective role in murine ALI. STAT6 signaling maybe a potential therapeutic target in murine ALI and patients with ARDS.

## Conclusions

In this study, we found that lack of STAT6 enhanced the development of ALI in mice, associated with increased macrophage pyroptosis and p38 MAPK signaling. Therefore, STAT6 signaling plays an immune regulatory role in murine ALI. STAT6 would be a potential therapeutic target in the treatment of ALI/ARDS.

## Supplementary Information

**Additional file 1: Fig. S1** Immunostaining for the expression of NLRP3, ASC and p-p38 MAPK in BMDMs. BMDMs from WT and STAT6 KO mice were treated with or without 500 ng/ml LPS for 24 hrs. The expression of NLRP3, ASC and p-p38 MAPK in the treated cells were analyzed by immunostaining. The cells were fixed with 4% paraformaldehyde, and followed by addition of 0.05% Triton-X 100 and 10% goat serum. The cells were then incubated with primary antibodies (dilution 200) for 3 hrs and followed by incubation with Cy3-conjugated secondary antibody for 1 hr (dilution 500). Red: positively stained cells. Blue: DAPI-stained nuclei. Representative photograph with 200 × magnification

**Additional file 2: Fig. S2** Uncropped full-length blots were included for Fig. 1D and Fig. 3C.20 µg protein samples were resolved on 15 wells and 1.5 mm thickness of 10% SDS-PAGE gel. After running for 1 h at 100 V, the resolved protein was transferred onto polyvinylidene fluoride membranes. The blots were then cut around the expected protein size, according to protein size marker and incubated with indicated primary antibodies. The blots were stripped for multiple hybridization. Images show full-length of original blots with visible membrane edges. In Fig. 1D, BMDMs from WT and STAT6 KO mice were treated with or without 500 ng/ml LPS for 24 hrs. The expression of total STAT6 in the treated cells were analyzed. GAPDH was internal loading control. In Fig. 3C, the expression of NLRP3, ASC, p-p38 MAPK, total p38 MAPK, p62 and LC3 in the lung tissues of mice with ALI was analyzed. GAPDH and β-Tubulin were internal loading controls. The lanes used in Fig. 1D and Fig. 3C were labeled

**Additional file 3: Fig. S3** Uncropped full-length blots were included for Fig. 5A and Fig. 5C.BMDMs from WT and STAT6 KO mice were treated with or without 500 ng/ml LPS for 24 hrs. In Fig. 5A, the expression of NLRP3 and p62 in the treated WT and KO BMDMs was analyzed. In Fig. 5C, the expression of p-p38 MAPK and total p38 MAPK in the treated BMDMs were analyzed. GAPDH was internal loading controls. The lanes used in Fig. 5A and Fig. 5C were labeled

## Data Availability

All data and materials were available from the corresponding author.

## Abbreviations

ALI: Murine acute lung injury
ARDS: Acute respiratory distress syndrome
ASC: Apoptosis-associated speck-like protein containing a CARD
BAL: Bronchoalveolar lavage
BMDMs: Bone marrow-derived macrophages
GAPDH: Glyceraldehyde-3-phosphate dehydrogenase
KO: Knock-out
LPS: Lipopolysaccharides
LC3: Microtubule-associated protein-1 light chain-3
p38 MAPK: P38 mitogen-activated protein kinase
MPs: Macrophages
NLRP3: Nod-like receptor containing a pyrin domain 3
NPs: Neutrophils
STAT6: Signal transducer and activator of transcription 6
WT: Wild-type
