# Supplementary figures and images for: Lack of STAT6 enhances murine acute lung injury through NLRP3/p38 MAPK signaling pathway in macrophages
Source: BMC Immunol. 2022 May 23;23:25. doi: 10.1186/s12865-022-00500-9 (PMC9126100; doi:10.1186/s12865-022-00500-9)

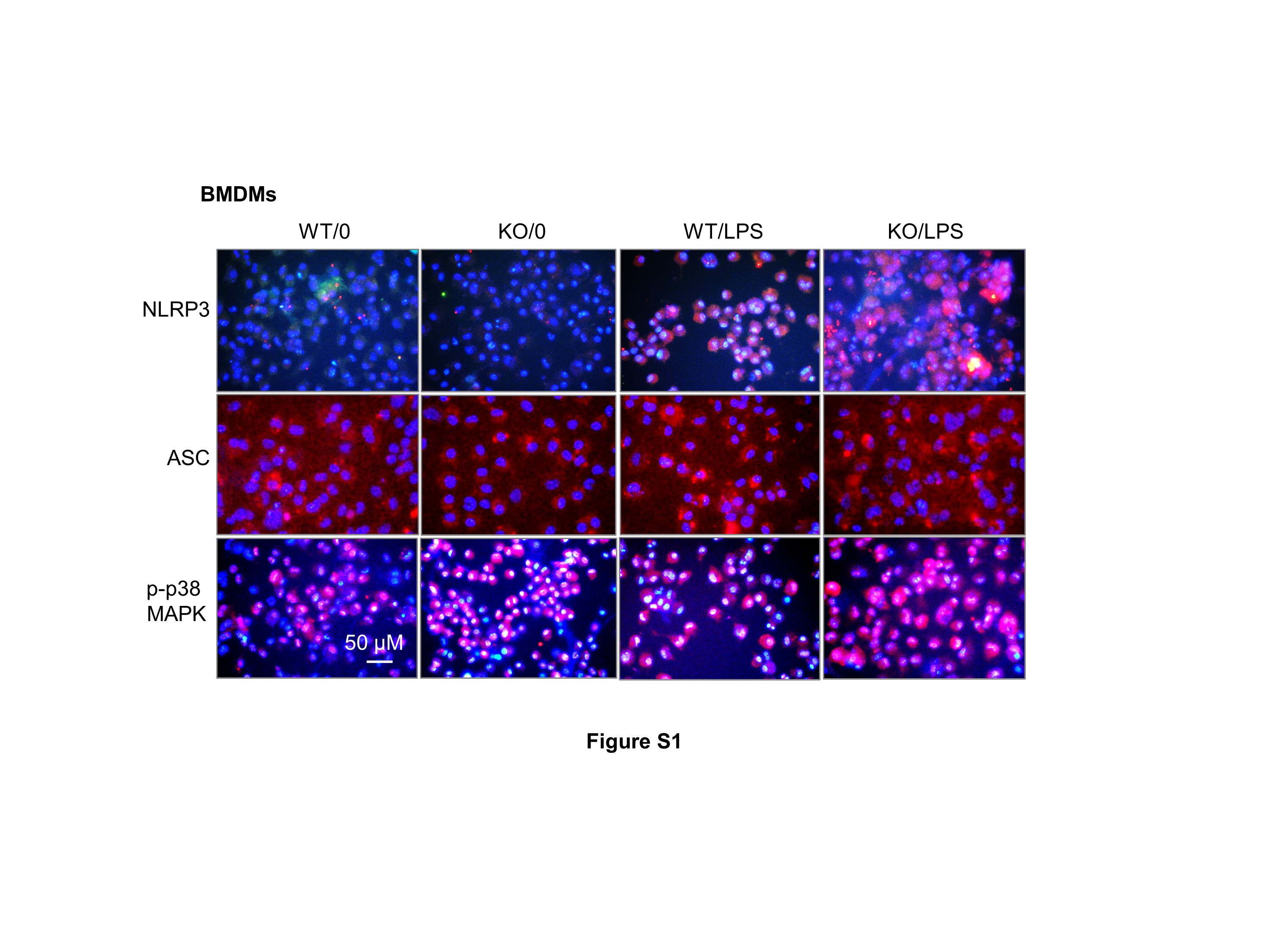

Supplement: Supplementary file 1 — Additional file 1: Fig. S1 Immunostaining for the expression of NLRP3, ASC and p-p38 MAPK in BMDMs. BMDMs from WT and STAT6 KO mice were treated with or without 500 ng/ml LPS for 24 hrs. The expression of NLRP3, ASC and p-p38 MAPK in the treated cells were analyzed by immunostaining. The cells were fixed with 4% paraformaldehyde, and followed by addition of 0.05% Triton-X 100 and 10% goat serum. The cells were then incubated with primary antibodies (dilution 200) for 3 hrs and followed by incubation with Cy3-conjugated secondary antibody for 1 hr (dilution 500). Red: positively stained cells. Blue: DAPI-stained nuclei. Representative photograph with 200 × magnification [file 12865_2022_500_MOESM1_ESM.jpg]

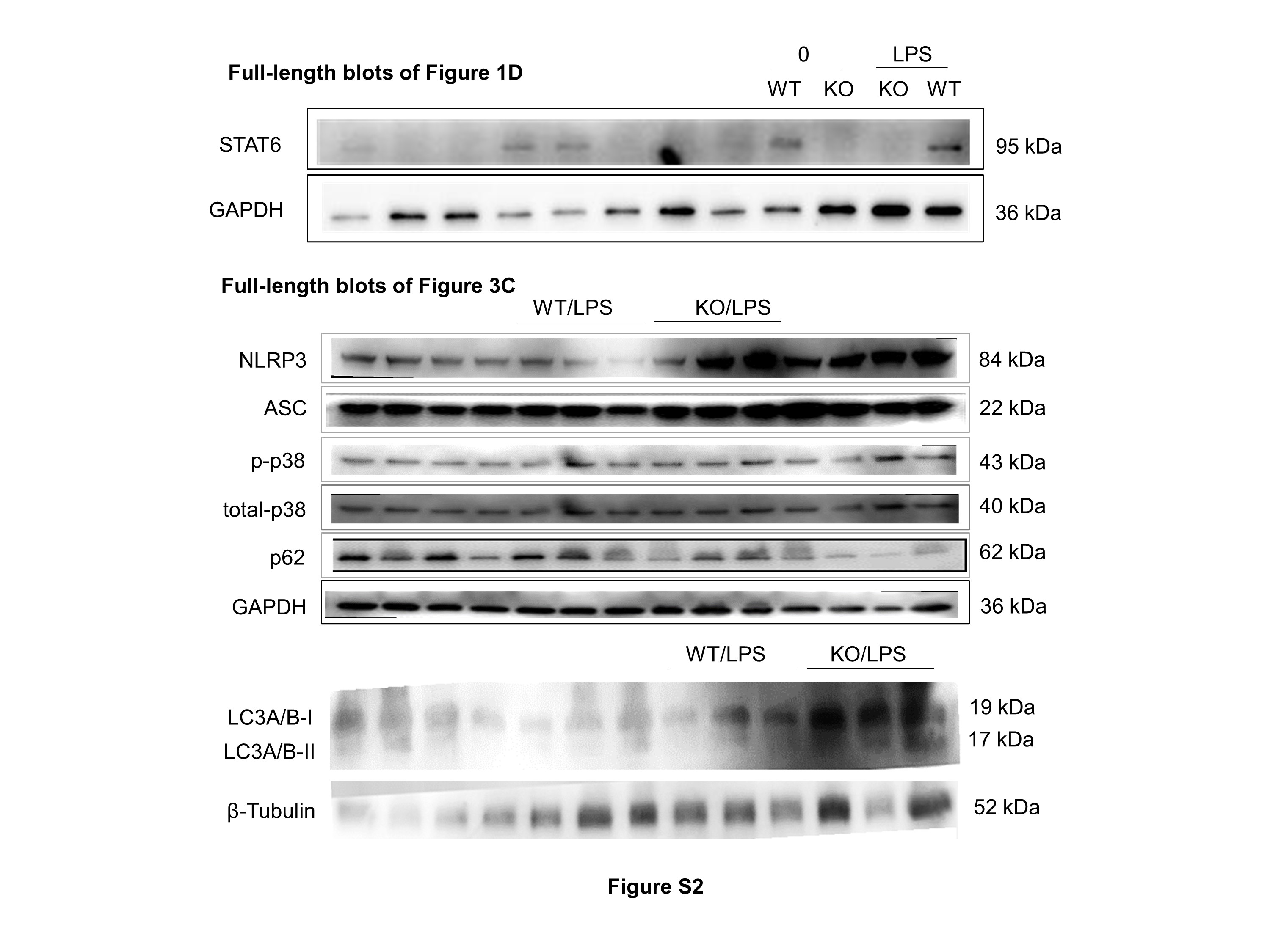

Supplement: Supplementary file 2 — Additional file 2: Fig. S2 Uncropped full-length blots were included for Fig. 1D and Fig. 3C.20 µg protein samples were resolved on 15 wells and 1.5 mm thickness of 10% SDS-PAGE gel. After running for 1 h at 100 V, the resolved protein was transferred onto polyvinylidene fluoride membranes. The blots were then cut around the expected protein size, according to protein size marker and incubated with indicated primary antibodies. The blots were stripped for multiple hybridization. Images show full-length of original blots with visible membrane edges. In Fig. 1D, BMDMs from WT and STAT6 KO mice were treated with or without 500 ng/ml LPS for 24 hrs. The expression of total STAT6 in the treated cells were analyzed. GAPDH was internal loading control. In Fig. 3C, the expression of NLRP3, ASC, p-p38 MAPK, total p38 MAPK, p62 and LC3 in the lung tissues of mice with ALI was analyzed. GAPDH and β-Tubulin were internal loading controls. The lanes used in Fig. 1D and Fig. 3C were labeled [file 12865_2022_500_MOESM2_ESM.jpg]

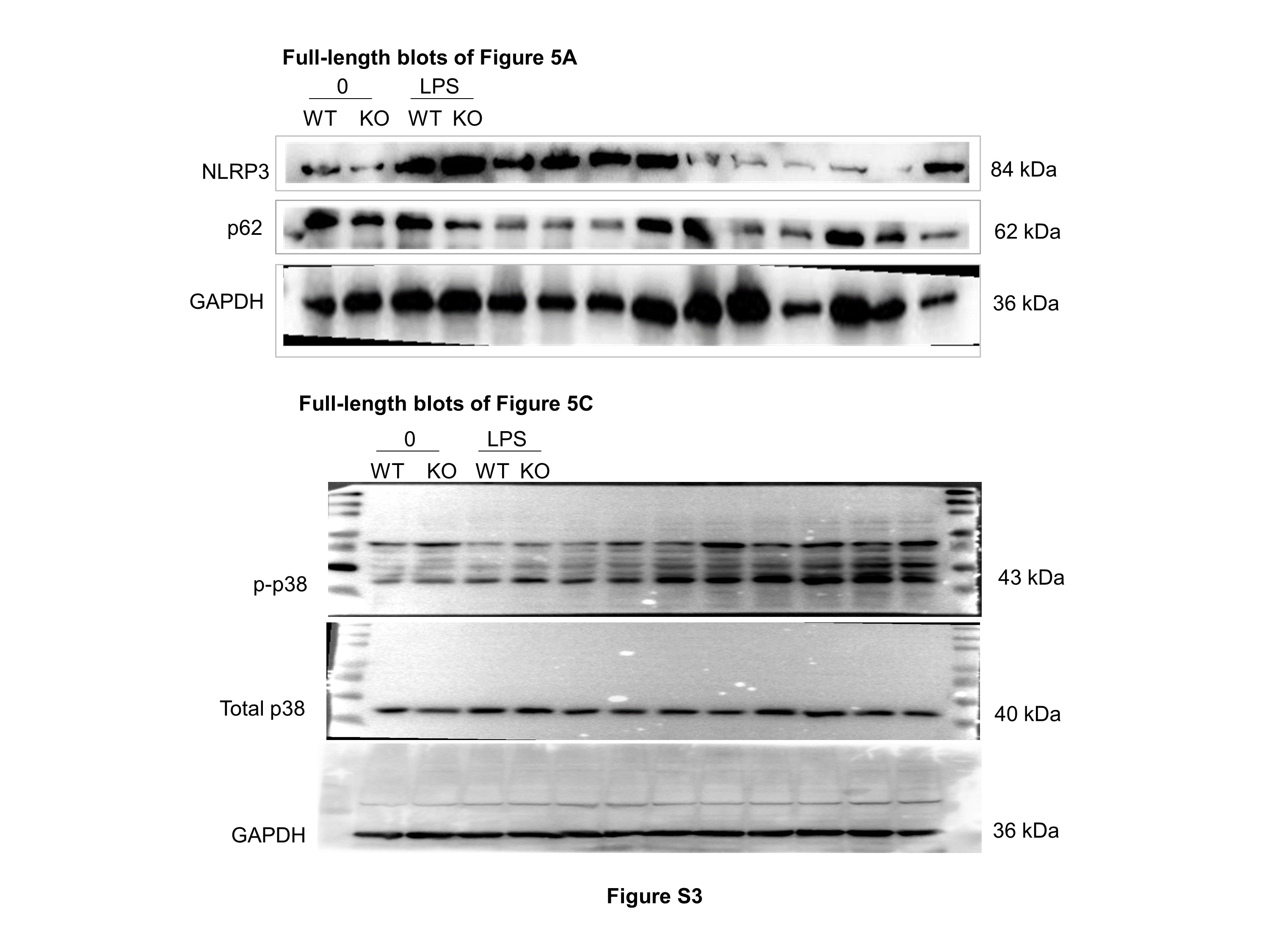

Supplement: Supplementary file 3 — Additional file 3: Fig. S3 Uncropped full-length blots were included for Fig. 5A and Fig. 5C.BMDMs from WT and STAT6 KO mice were treated with or without 500 ng/ml LPS for 24 hrs. In Fig. 5A, the expression of NLRP3 and p62 in the treated WT and KO BMDMs was analyzed. In Fig. 5C, the expression of p-p38 MAPK and total p38 MAPK in the treated BMDMs were analyzed. GAPDH was internal loading controls. The lanes used in Fig. 5A and Fig. 5C were labeled [file 12865_2022_500_MOESM3_ESM.jpg]
